# High Reoperation Rate in Mobile-Bearing Total Ankle Arthroplasty in Young Patients

**DOI:** 10.3390/medicina58020288

**Published:** 2022-02-14

**Authors:** Christian Stadler, Matthias Luger, Stella Stevoska, Manuel Gahleitner, Lorenz Pisecky, Tobias Gotterbarm, Antonio Klasan, Matthias C. Klotz

**Affiliations:** 1 Department for Orthopaedics and Traumatology, Med Campus III, Kepler University Hospital GmbH, Johannes Kepler University Linz, Krankenhausstr. 9, 4020 Linz, Austria; matthias.luger@kepleruniklinikum.at (M.L.); stella.stevoska@kepleruniklinikum.at (S.S.); manuel.gahleitner@kepleruniklinikum.at (M.G.); lorenz.pisecky@kepleruniklinikum.at (L.P.); tobias.gotterbarm@kepleruniklinikum.at (T.G.); antonio.klasan@kepleruniklinikum.at (A.K.); 2 Marienkrankenhaus Soest, Orthopaedics and Trauma Surgery, Widumgasse 5, 59494 Soest, Germany; m.klotz@hospitalverbund.de

**Keywords:** total ankle arthroplasty, ankle replacement, ankle arthritis, endoprosthetics, arthrodesis, salto mobile-bearing

## Abstract

*Background and Objectives:* Due to inferior survival rates compared to hip and knee arthroplasty, total ankle arthroplasty (TAA) was previously mainly recommended for older and less active patients. However, given the encouraging survival rates and clinical outcomes of modern generations of TAA, some authors have also advocated TAA in young patients. Thus, the aim of this study was to evaluate age related reoperation, revision and survival rates of third-generation mobile-bearing TAAs. *Materials and* *Methods:* In this retrospective study, 224 consecutive TAA patients with a minimum follow up (FU) of 2 years were analyzed. Patients were retrospectively assigned to two study groups (Group A: age < 50 years; Group B: age ≥ 50 years). Revision was defined as secondary surgery with prothesis component removal, while reoperation was defined as a non-revisional secondary surgery involving the ankle. *Results:* After a mean FU of 7.1 ± 3.2 years, the reoperation rate (Group A: 22.2%; Group B: 5.3%; *p* = 0.003) and revision rate (Group A: 36.1%; Group B: 13.8%; *p* = 0.003) were higher within Group A. An age of under 50 years at time of surgery was associated with higher reoperation (odds ratio (OR): 6.54 (95% CI: 1.96–21.8); *p* = 0.002) and revision rates (OR: 3.13 (95% CI: 1.22–8.04); *p* = 0.018). Overall, lower patient age was associated with higher reoperation (*p* = 0.009) and revision rates (*p* = 0.001). *Conclusions:* The ideal indication for TAA remains controversial, especially regarding patient age. The findings of this study show high reoperation and revision rates in patients aged under 50 years at time of surgery. Therefore, the outcomes of this study suggest that the indication for TAA in young patients should be considered very carefully and that the association between low patient age and high reoperation rate should be disclosed to all eligible patients.

## 1. Introduction

While arthroplasty is the gold standard for the treatment of end-stage osteoarthritis (OA) in the hip and knee, arthrodesis has been the treatment of choice for the ankle in the past [1,2]. In recent years, previous studies have reported encouraging survival rates for newer generations of total ankle arthroplasties (TAAs), so the arthroplasty of the ankle is gaining in popularity [3,4,5,6,7,8,9]. Nevertheless, the decision between TAA and ankle arthrodesis has to be carefully considered for each patient individually—especially in case of severe instability or deformity [10]. The intended advantages of TAA are good residual mobility and thus improved function compared to arthrodesis [11,12]. In addition, an improved mobility of the ankle joint is intended to reduce the risk of subsequent OA in adjacent joints [11,12,13,14,15,16]. Due to its inferior survival rates compared to hip and knee replacement, TAA was mainly recommended for older and less active patients in the past [17,18,19]. However, some recent publications have advocated for various TAA implants in young patients [17,18,19,20,21,22,23]. Gaugler et al. and Lee et al. reported no significant impact of age at time of surgery on the prothesis survival or reoperation rates for Hintegra TAA (Allegra Orthopaedics, Sydney, NSW, Australia) [17,20]. Rodrigues-Pinto et al. analyzed the age-related outcomes of the Salto mobile-bearing TAA (Tornier SA, Saint Ismier, France) within a mean follow up (FU) of 3.4 years and found no significant age-related differences in complication and prothesis survival rates [18]. Currently, there are no reports available regarding age-related reoperation rates and survival for the Salto mobile-bearing TAA with a mean FU of more than 5 years and a large study population.

Thus, the aim of this study was to evaluate the age-related revision, reoperation, implant survival rates of the Salto mobile-bearing TAA with a long-term FU in a large cohort.

## 2. Materials and Methods

### 2.1. Study Type and Population

**Figure 1:**
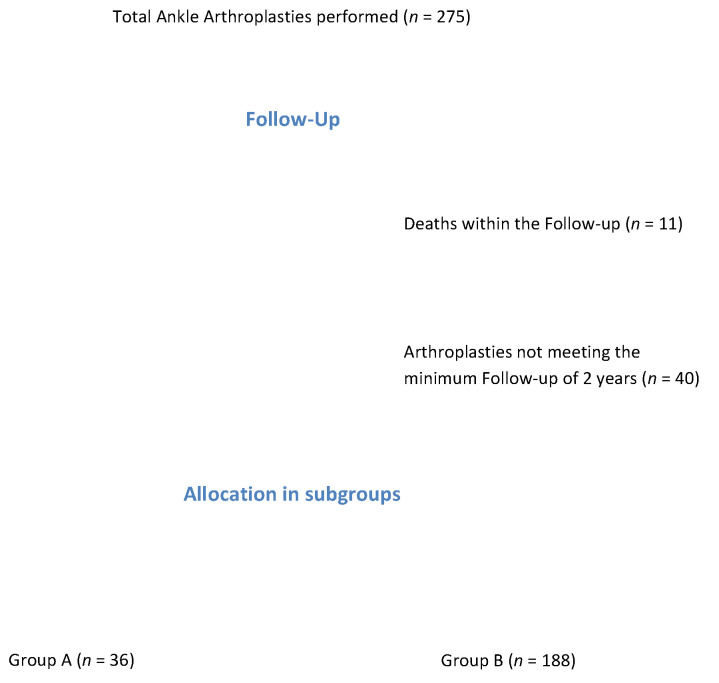
Flowchart regarding formation of the study population and its allocation in the two study subgroups dependent on the patient’s age at time of surgery (Group A = age under 50 years; Group B = age above 50 years).

This was a retrospective cohort study. Inclusion criteria for this study were the implantation of a third-generation Salto mobile-bearing TAA (Tornier SA, Saint Ismier, France) between March 2002 and November 2015 with a minimum follow up (FU) of 2 years. A total of 275 TAAs were consecutively implanted within the time period mentioned above by two senior orthopedic surgeons, each with more than 10 years of experience in foot and ankle surgery. Within the FU period, a total of 11 patients died and 40 protheses did not meet the inclusion criteria mentioned above, resulting in an overall study population of 224 TAAs. The study population was divided into two subgroups retrospectively according to the patient’s age at time of surgery. Patients aged under 50 years were assigned to Group A, and patients aged 50 years or above were assigned to Group B (Figure 1) [18]. Contraindications for TAA were physically demanding professions, excessive sports on a regular basis, severe neurological disorders, uncontrolled diabetes mellitus, poor peripheral blood circulation, inadequate bone stock, severe instability, and history of recent ankle infection [24,25]. The study center was a university hospital in Austria.

### 2.2. Preoperative Preparation, Surgical Technique, Postoperative Care and Follow up

Preoperatively, a thorough clinical examination of the patient and the ankle was performed by the surgeon. Prior to skin incision, antibiotic prophylaxis was administered as an intravenous single shot (1.5 g of cefuroxime followed by 600 mg of clindamycin for patients with a penicillin allergy). The Salto mobile-bearing TAA was implanted using an anterior approach to the ankle’s articular capsule between the M. tibialis anterior’s and the M. extensor hallucis longus’ tendon. After chiseling surrounding osteophytes (if present), the components of the prothesis were implanted according to the manufacturer’s instructions [25,26]. After performing the TAA, an adequate range of motion (ROM) of the ankle joint was intraoperatively checked prior to wound closure. A short leg cast was applied to every patient for a total of six weeks after finishing surgery in the operation room immediately after wound closure and sterile wound dressing. No weightbearing was recommended to every patient for two weeks, followed by partial weightbearing for another two weeks and full weightbearing for the last two weeks prior to the removal of the short leg cast. Venous thrombosis prophylaxis was postoperatively ensured throughout the entire first six weeks.

Physiotherapy was provided to every patient from day one after surgery to instruct the correct usage of crutches and to mobilize patients, train coordinative skills, and prevent excessive muscular atrophy of the not immobilized lower extremities. Patients stayed an average of 9.4 ± 3.2 days at the hospital and were discharged once adequate local conditions at the operated ankle including swelling and wound status were achieved. Patients were checked up at the outpatient clinic two weeks after surgery for suture removal and the application of a circulated short leg cast. Further check-ups at the outpatient clinic were recommended to every patient four weeks, six weeks, three months, and one year after surgery. After the first postoperative year, additional check-ups were recommended after every two years.

Following the removal of the short leg cast, physiotherapy was suggested to every patient in order to improve the ankle’s ROM as effectively as possible and to counteract muscular atrophy caused by the postoperative immobilization of the operated leg. Full weight bearing was permitted after the removal of the short leg cast, but the avoidance of physical activities with high impact on the ankle was recommended to every patient.

Patient records were retrospectively screened for complications that led to secondary surgery. As for the secondary surgeries following the primary TAA, the definitions introduced by Henricson et al. were applied [27]. Therefore, revision was defined as secondary surgery with the removal of at least one of the prothesis’ components and reoperation was defined as secondary surgery of the ankle without the removal of one of the prothesis’ components except for an incidental exchange of the polyethylene inlay. Secondary surgeries related to the TAA but not involving the actual joint were defined as additional procedures.

End of follow up was defined as the date of secondary surgery or as the date of the last check-up at the outpatient clinic if no adverse event with subsequent reoperation occurred.

### 2.3. Statistical Analysis

SPSS (version 27.0, IBM, Armonk, NY, USA) was used for the statistical analysis. The Kolmogorov–Smirnov test was performed to test for normality distribution. For metric-scaled data, the arithmetic mean and standard deviation were calculated, and these two parameters are reported as arithmetic mean value ± standard deviation. Kruskal–Wallis was used for the evaluation of differences between the study groups regarding non-normally distributed parameters, and the *t*-test was used to analyze normally distributed parameters. The difference between nominally scaled parameters was analyzed using the chi square test. The survival analysis of the TAA was conducted via Kaplan–Meier analysis including the log rank test, with any reoperation and revision as the endpoint.

The analysis of associations between the patient characteristics and the rate of reoperations and revisions within the two study groups as dependent variables was performed with a binary logistic regression.

The level of significance was defined at *p* ≤ 0.05.

## 3. Results

### 3.1. Demographic Characteristics

The mean FU within the study population was 7.1 ± 3.2 years. The mean age within whole the study population was 61.4 ± 11.8 years, and it was 41.4 ± 6.7 years in Group A and 65.2 ± 8.3 years in Group B. The detailed characteristics of the study population and the indications for TAA are shown in Table 1.

### 3.2. Complications and Revisions

Within the follow-up period, secondary surgery due to complications related to TAA including revisions, reoperations and additional procedures was performed in 60 patients (26.7%). The detailed numbers regarding the complications that led to secondary surgery are shown in Table 2, and the types of the performed procedures are shown in Table 3. In 44 cases, more than one complication was addressed during the performed secondary surgery. In a total of six cases, the first revision surgery failed and was followed by the removal of the TAA. Following the removal of the TAA (*n* = 16), arthrodesis was performed in 14 cases (9 nail arthrodeses addressing the ankle joint and subtalar joint and 5 plate arthrodeses only addressing the ankle joint), and a revision TAA was performed in 2 cases.

In two cases, the indication for secondary surgery could not be determined because the procedure was performed in another hospital; in one case, no detailed information regarding the performed revisional surgery was available because it was performed in another hospital.

According to the definition of reoperation and revision mentioned above [27] the reoperation rate was 22.2% (8 reoperations within 36 ankles) within Group A and 5.3% (10 reoperations within 188 ankles) within Group B (*p* = 0.003). The reoperation rate within the whole study population was 8.0% (18 reoperations within 224 ankles).

The revision rate was 36.1% (13 revisions within 36 ankles) within Group A and 13.8% (26 revisions within 188 ankles) within Group B (*p* = 0.003). The revision rate within the whole study population was 17.4% (39 revisions within 224 ankles).

Within Group A, the rate of all secondary procedures including revisions, reoperations and additional procedures related to TAA was 58.3% (21 secondary procedures within 36 ankles), and it was 26.2% (39 secondary procedures within 149 ankles) within Group B (*p* < 0.001). The overall rate of secondary procedures within the whole study population was 26.8% (60 secondary procedures within 224 ankles).

### 3.3. Survival Analysis and Regression Analysis

**Figure 2:**
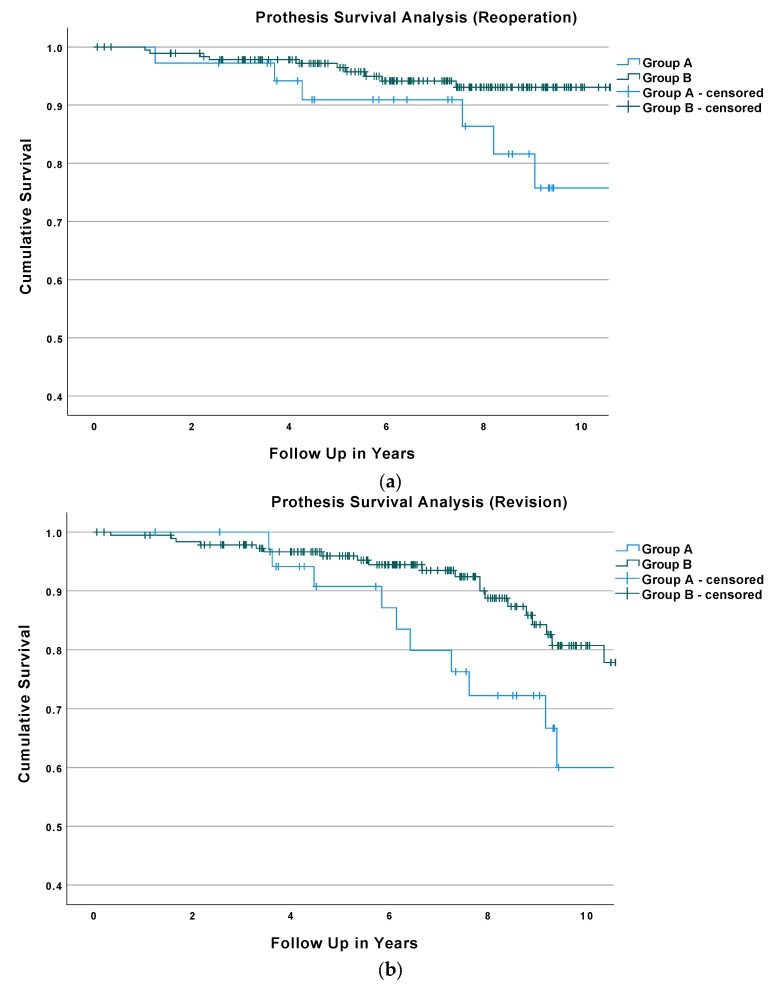

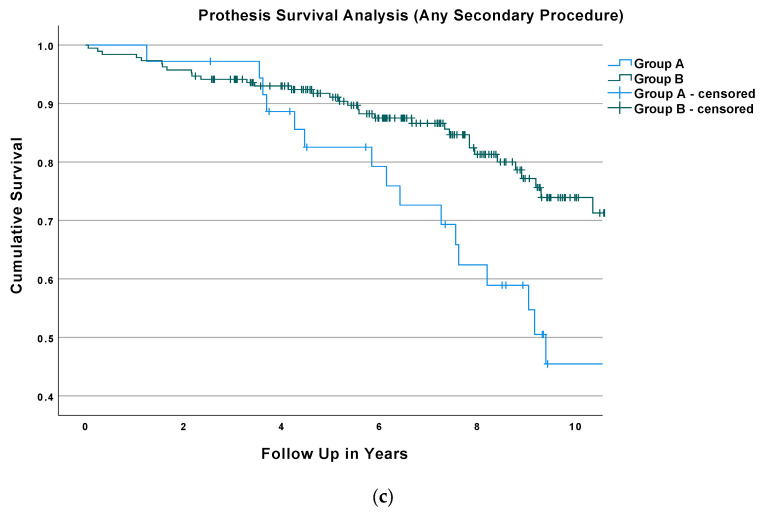
(**a**). Results of the Kaplan–Meier Survival analysis of both study groups with reoperation as the endpoint. (**b**) Results of the Kaplan–Meier Survival analysis of both study groups with revision as the endpoint. (**c**) Results of the Kaplan–Meier Survival analysis of both study groups with any secondary surgery as the endpoint.

The Kaplan–Meier survival analysis with reoperation as the endpoint showed that the 5-year survival rate was 90.9% ± 5.0% for Group A and 96.5% ± 1.4% for Group B. The 7-year survival rate was 90.9% ± 5.0% for Group A and 94.2% ± 1.9% for Group B; see Figure 2a. The difference between the prothesis survival of Groups A and B with reoperation as the endpoint was significant according to the performed log rank test (*p* = 0.007).

The Kaplan–Meier survival analysis with revision as the endpoint revealed a 5-year survival rate of 90.8% ± 5.1% for Group A and 96.0% ± 1.5% for Group B. The 7-year survival rate was 79.9% ± 7.4% for Group A and 93.5% ± 2.0% for Group B; Figure 2b. The difference between the prothesis survival of Groups A and B with revision as the endpoint was significant according to the performed log rank test (*p* = 0.036).

The Kaplan–Meier survival analysis with any secondary procedure as the endpoint revealed a 5-year survival rate of 82.5% ± 6.5 for Group A and a 5-year survival rate of 91.1% ± 2.1% for Group B. The 7-year survival rate was 72.6% ± 7.8% for Group A and 86.6% ± 2.7%for Group B; see Figure 2c. The difference between the prothesis survival rate with any secondary surgery as the endpoint of Groups A and B was significant according to the performed log rank test (*p* = 0.003).

The binary logistic regression performed within the study population revealed an odds ratio of 6.54 (95% CI: 1.96–21.8; *p* = 0.002) for reoperations, an odds ratio of 3.13 (95% CI: 1.22–8.04; *p* = 0.018) for revision, and an odds ratio of 5.35 (95% CI: 2.24–12.80; *p* > 0.001) for any secondary procedure for Group A. The binary logistic regression performed on patient characteristics within the whole study population revealed an odds ratio for every additional year of age of 0.94 (95% CI: 0.90–0.99; *p* = 0.009) for reoperations, an odds ratio for every additional year of age of 0.94 (95% CI: 0.90–0.97; *p* = 0.001) for revisions, and an odds ratio for every additional year of age of 0.93 (95% CI: 0.90–0.96; *p* > 0.001) for any secondary procedures. No other analyzed patient characteristics showed a significant effect on the reoperation or revision rates. See Table 4 for the detailed results of the logistic regression.

## 4. Discussion

The results of this study revealed a significantly higher rate of secondary procedures related to TAA for patients aged under 50 years at time of surgery compared to patients aged 50 years or above at time of surgery. The overall secondary procedure rate was 58.3% in Group A and 26.8% in Group B (less than half compared to Group A). Additionally, the binary logistic regression performed within the whole study population independently from the patients’ allocation to the two study groups showed a significant effect of age on the rate of secondary procedures within the FU.

These findings confirm that the prevalent opinion of some authors, who have described the ideal patient eligible for TAA as relatively old with low physical demands [10,28,29,30], can also be applied for modern TAA implants. Similar to other joint arthroplasties, such as total hip arthroplasty and total knee arthroplasty, young patient age has been found to be associated with an increased revision rate many times [31,32,33,34,35].

Nevertheless, some recent studies regarding modern TAA have questioned the indication of TAA in young patients. Satisfying outcomes including implant survival and reoperation rates with no significant difference to older patients have been more often reported recently [17,18,19,20,22,23], which is clearly in contrast to the findings of this study. For example, a retrospective cohort study conducted by Gaugler et al. using the Hintegra implant in a cohort of 811 patients revealed no significant effect of age on the rates for minor or major revisions, while the clinical outcomes of younger and older patients were comparable with slightly better pain relief in older patients [20]. Similarly, Lee et al. reported no significant difference in revisions rates and clinical outcomes related to age at time of surgery when using the Hintegra implant in a cohort of 117 patients [17].

Demetracopoulos et al. prospectively analyzed several different TAA implants and found no significant difference regarding complication and reoperation rates related to age at time of surgery in a cohort of 395 patients [22]. Rodrigues-Pinto et al., who investigated the same TAA implant as the present study, conducted a prospective multicentric study in a cohort of 103 patients and found no significant differences in revision rates and implant survival between patients aged below or above 50 years at time of surgery, while significantly better clinical outcomes were found postoperatively in patients aged below 50 years at time of surgery [18]. Cottom et al. investigated several different implants in 112 patients and reported the following complication rates: 18% for patients younger than 55 years, 11.6% for patients aged between 55 and 70 years, and 9.4% for patients older than 70 years at time of surgery. At a mean follow up of 33.9 months, those differences regarding complication rates were not statistically significant [19].

Apart from Gaugler et al. (811 TAAs; 5.4–6.9 years mean FU), the studies mentioned above featured either a shorter mean FU or a smaller study population (Lee et al.: 6.5 years and 117 TAAs; Demetracopoulos et al.: 3.5 years and 395 TAAs; Rodrigues-Pinto et al.: 3.4 years and 103 TAAs; and Cottom et al.: 2.8 years and 112 TAAs). The length of the mean FU seems to be an important factor in age-related revision rates after TAA, as within the survival analysis of our study population, the survival rate between the two groups was very much alike until approximately 4 years after surgery, with a notable greater decline of the survival rate of Group A following the 4th year after surgery (Figure 2a–c). Additionally, except for that of Rodrigues-Pinot et al., the studies mentioned above investigated different implants than this study. All of these differences complicate the comparability between these studies and may explain the heterogenous age-related outcomes and reoperations rates reported in the literature.

All the studies mentioned above investigated a possible association between patient age at time of surgery and complication rate. Although some of those studies—such as the study conducted by Cottom et al. [19]—implied tendencies towards higher complication rates in young patients, none of those studies found a statistically significant association between patient age at time of surgery and complication rate.

Due to the study design, we were not able to determine causal reasons for the higher rate of complications and secondary procedures of younger patients within the study population. However, one factor contributing to the higher complication rate might be an averagely higher physical demand and more intensive physical activities of younger patients, which might cause the accelerated wear of prothesis’ components and increased periarticular soft tissue and bone affection [36,37,38,39,40]. Due to the study design and the limitations mentioned below, we were also not able to reliably comment on the occurrence of OA of adjacent joints after TAA. Due to residual mobility, OA of adjacent joints should in theory occur less often after TAA than after ankle arthrodesis [11,12]. However, there is a lack of studies directly comparing OA of adjacent joints after TAA and ankle arthrodesis, such as in a prospective randomized controlled trial setting [13,14,15,16]. Additionally, different rates of OA of adjacent joints after ankle arthrodesis have been reported in the literature [41]. Additionally, there seem to be differences between different types of ankle arthrodeses, e.g., Morasiewicz et al. found a lower rate of OA in adjacent joints after using an external Ilizarov fixation for ankle arthrodesis compared to an internal fixation for ankle arthrodesis [42]. To further evaluate this matter, more studies with appropriate study designs are necessary.

This study had several limitations that must be kept in mind when interpreting its findings. The retrospective study design was a major limitation, as no causal conclusions could be drawn from the results. Additionally, no clinical outcome measures or radiographic outcomes were analyzed within this study. Therefore, we were not able to comment on the clinical and radiological outcomes including patient satisfaction within the study population. For future investigations, a prospective study design including patient-reported outcome measures would be more appropriate to thoroughly analyze the effects of certain patient characteristics, such as physical activity, on the revision rates and overall outcomes after TAA. A total of 40 protheses could not be included in the study due to a lack of the minimum FU of 2 years, which represents another limitation, as does the unequal distribution of the number of patients between the two study groups. One possible explanation for the unequal distribution might be the higher prevalence of OA in older patients in general [43,44]. Furthermore, it was not possible to retrospectively determine the duration of each surgery. Therefore, we were not able to comment on a possible influence of duration of surgery on the outcome, specifically the reoperation and revision rates. 

## 5. Conclusions

The ideal indication for TAA remains controversial, especially when it comes to the treatment of patients aged under 50 years. The findings of this study show a high reoperation rate in young patients. More than half of young patients underwent secondary surgery within a mean FU of 7 years within our study population. Therefore, the outcomes of this study suggest that the indication for TAA in patients aged under 50 years should be considered very carefully and that the association between low patient age and high reoperation rate should be disclosed to all eligible patients.

## Figures and Tables

**Table 1:** Baseline characteristics of the two study groups including the indications for TAA.

Baseline Characteristics of the Study Population	Overall (*n* = 224)	Group A (*n* = 36)	Group B (*n* = 188)	*p*-Value
Mean follow up (years)	7.1 ± 3.2	7.8 ± 3.5	7.0 ± 3.1	0.187
Mean age (years)	61.4 ± 11.8	41.7 ± 6.7	65.2 ± 8.3	<0.001
Female	107	13	94	0.147
Male	117	23	94	0.147
Right Ankle	130	22	108	0.716
Left Ankle	94	14	80	0.716
Mean body weight (kg)	80.9 ± 17.0	83.5 ± 13.9	80.4 ± 17.5	0.318
Mean body height (cm)	169.6 ± 14.7	174.4 ± 8.1	167.6 ± 18.9	0.002
Mean BMI	28.0 ± 4.3	27.4 ± 3.7	28.2 ± 4.4	0.312
Mean ASA Score	1.9 ± 0.5	1.5 ± 0.6	2.0 ± 0.5	<0.001
Mean size tibial component	1.9 ± 0.8	2.0 ± 0.8	1.9 ± 0.8	0.412
Mean size talar component	1.6 ± 0.6	1.7 ± 0.6	1.5 ± 0.6	0.140
Mean size polyethylene inlay	5.5 ± 1.1	5.3 ± 1.2	5.5 ± 1.1	0.237
**Indications for Total Ankle Arthroplasty**	**Overall** **(*n* = 224)**	**Group A** **(*n* = 36)**	**Group B** **(*n* = 188)**	
Posttraumatic osteoarthritis	144	27	117	0.143
Primary osteoarthritis	53	1	52	0.001
Chronic inflammatory diseases	16	4	12	0.313
Prior infection	5	2	3	0.016
Aseptic osteonecrosis	3	2	1	0.141
Haemochromatosis	3	0	3	0.445

**Table 2:** Detailed numbers regarding the complications that led to secondary surgery. In a total of 35 cases, two or more of the complications listed below led to secondary surgery.

Complication	Group A (*n* = 21)	Group B (*n* = 39)	Overall (*n* = 60)
Osteolytic cysts	10	10	20
Inlay fracture	6	13	19
Ossifications	10	7	17
Wear	5	6	11
Soft tissue impingement	3	5	8
Contracture/ROM-Limitation	3	2	5
Acute infection	0	4	4
Deep wound infection	0	2	2
Inlay luxation	0	2	2
Aseptic osteonecrosis	0	1	1
Talonavicular OA	0	1	1
Instability	0	1	1
Achilles’ tendon rupture	0	1	1
No information available	1	1	2

**Table 3:** Detailed numbers regarding the types of procedures performed to address the complications related to TAA mentioned above. In a total of 44 cases, one or more procedures were performed within the secondary surgery.

	Group A (*n* = 21)	Group B (*n* = 39)	Overall (*n* = 60)
Inlay replacement	18	27	45
Synovectomy	10	14	24
Filling of osteolytic cysts	9	8	17
Removal of ossifications	10	7	17
Explantation	6	10	16
Arthrodesis	5	9	14
Revision prothesis	1	1	2
Achilles’ tendon lengthening	2	2	4
Corrective osteotomy	0	3	3
Ligamentous release	1	1	2
Lateral ligament repair	0	1	1
Achilles’ tendon repair	0	1	1
Talonavicular arthrodesis	0	1	1
Flap Surgery	0	1	1
Wound revision	0	1	1
Subtalar arthrodesis	0	1	1
No information available	0	1	1

**Table 4:** Results of the binary logistic regression reporting the odds ratio for reoperation, revision, and any secondary procedure of each patient characteristic with the 95% CI and level of significance in brackets.

Characteristic	Reoperation	Revision	Any Sec. Procedure
Age (years)	0.94 (0.90–0.99; *p* = 0.009)	0.94 (0.90–0.97; *p* = 0.001)	0.93 (0.90–0.96; *p* > 0.001)
Sex (male)	1.38 (0.32–5.90; *p* = 0.664)	1.24 (0.42–3.68; *p* = 0.705)	1.25 (0.49–3.20; *p* = 0.639)
Body height (cm)	1.09 (0.70–1.70; *p* = 0.703)	1.01 (0.92–1.18; *p* = 0.796)	1.02 (0.92–1.14; *p* = 0.698)
Body weight (kg)	0.89 (0.56–1.42; *p* = 0.603)	1.01 (0.90–1.15; *p* = 0.838)	0.99 (0.87–1.12; *p* = 0.830)
Body Mass Index	1.27 (0.33–4.93; *p* = 0.726)	0.94 (0.65–1.36; *p* = 0.747)	0.98 (0.68–1.42; *p* = 0.918)
ASA-Score	1.23 (0.47–3.20; *p* = 0.678)	1.48 (0.69–3.18; *p* = 0.320)	1.27 (0.65–2.48; *p* = 0.490)

## Data Availability

The data presented in this study are available on request from the corresponding author.
